# Evaluation of Postoperative Radiotherapy Effect on Survival of Resected Stage III-N2 Non-small Cell Lung Cancer Patients

**DOI:** 10.3389/fonc.2020.01135

**Published:** 2020-07-28

**Authors:** Fei Gao, Nan Li, YongMei Xu, GuoWang Yang

**Affiliations:** ^1^Department of Oncology & Hematology, Beijing Hospital of Traditional Chinese Medicine, Capital Medical University, Beijing, China; ^2^Graduate School, China Academy of Chinese Medical Sciences, Beijing, China

**Keywords:** non-small cell lung cancer, stage IIIA-N2, postoperative radiotherapy, overall survival, SEER database

## Abstract

**Objective:** The role of postoperative radiotherapy (PORT) in resected stage IIIA-N2 non-small cell lung cancer (NSCLC) patients remains controversial. This study aimed to explore the effect of PORT on survival of resected stage IIIA-N2 NSCLC patients.

**Methods:** Resected stage IIIA-N2 NSCLC patients aged 18 years or older were identified from the SEER (Surveillance, Epidemiology, and End Results) database from 2010 to 2015. Cox regression analysis was used to identify factors including PORT associated with survival time. A subgroup analysis of patients stratified by number of lymph node metastases was also performed. Overall survival (OS) and overall mortality were compared among the different groups.

**Results:** A total of 3,445 patients were included in the study. Multivariate Cox analysis showed that PORT had no significant impact on survival of patients with <6 positive lymph node [hazard ratio (HR) = 1.012, *P* = 0.858, 95% CI: 0.886–1.156]. Postoperative chemotherapy (POCT) (HR = 0.605, *P* < 0.001, 95% CI: 0.468–0.783) and PORT (HR = 0.724, *P* = 0.007, 95% CI: 0.574–0.914) are both favorable prognostic factors for stage IIIA-N2 patients with ≥6 positive lymph nodes. In 2,735 patients who featured <6 number of positive regional lymph nodes, patients who received PORT had better survival and lower 3-years and 5-years overall mortality rate than patients who underwent surgery only (41 vs. 28 months, *P* < 0.015). There was no significant difference in the survival of postoperative patients who underwent POCT in view of whether received PORT (44 vs. 53 months, *P* = 0.176). A total of 710 patients who featured ≥6 number of positive regional lymph node metastasis were divided into two groups by PORT. PORT did not prolong survival for postoperative patients who did not receive chemotherapy (12 vs. 15 months, *P* = 0.632). PORT showed a significant advantage in influencing OS in patients who received PORT combined with POCT as compared with those who received POCT only (32 vs. 25 months, *P* = 0.006).

**Conclusions:** For IIIA-N2 patients with <6 lymph node metastases, use of PORT can be encouraged to improve survival. For patients with ≥6 positive lymph nodes, PORT combined with POCT significantly improved OS and decreased overall mortality.

## Introduction

Lung cancer is one kind of the most frequent malignant tumors with the highest morbidity and mortality in the world. Non-small cell lung cancer (NSCLC) is the most common type, accounting for 80–85% of lung cancer (1), among which stage IIIA-N2 patients account for about 20% (2, 3). The benefit of radical surgery is limited for stage IIIA-N2 patients. Previous studies have shown that the 5-years survival rate of patients with stage IIIA-N2 NSCLC after lung cancer radical pneumonectomy was only 15–20% (4). The main cause of postoperative failure was local recurrence or distant metastasis (5, 6). Nearly 40% of patients have local recurrence or regional lymph node metastasis within 5 years after surgery, even if after a complete resection of lung cancer. Therefore, complete surgical resection combined with postoperative adjuvant therapy is still the main treatment mode for stage IIIA-N2 NSCLC patients.

Clinical evidence showed that postoperative chemotherapy (POCT) could improve the long-term survival of patients (7, 8). However, it has been reported that the local failure rate still exists despite complete resection and postoperative adjuvant chemotherapy. Local recurrence indicated the importance of local postoperative adjuvant therapy. Postoperative radiotherapy (PORT), as a kind of local treatment, can theoretically improve the local control rate and improve the survival of patients. However, whether PORT can improve the survival rate of stage IIIA-N2 NSCLC remains controversial. The selection of stage IIIA-N2 patients who can benefit from PORT is confusing for clinicians. Prospective randomized controlled studies of PORT for resected stage IIIA-N2 NSCLC patients are mostly single-center, small-sample-size studies, the radiotherapy technology used is old, and the radiation dose and range are not uniform, which will reduce the effectiveness of the existing clinical evidence. Our study was based on a population-based cohort to provide more evidence for the application of PORT in resected stage IIIA-N2 NSCLC patients.

## Materials and Methods

This retrospective study, which was approved by Ethics Committee of Beijing Hospital of Traditional Chinese Medicine, Capital Medical University, retrieved data from the SEER (Surveillance, Epidemiology, and End Results) database using SEER^*^STAT 8.3.6 software. Permission to access the custom data file in the SEER program was obtained, and the reference number was 14026-Nov2018. The SEER database, which aimed to reduce the cancer burden in Americans, recorded the incidence, mortality, and morbidity of millions of cancer patients in the United States over the past 40 years. At present, the number of registration stations has expanded to 18. These registration stations operated with the SEER^*^STAT software, a powerful computer tool for statistical analysis, and submitted data to NCI twice a year for classification, statistics, and aggregation.

We extracted data of lung cancer patients registered from 2010 to 2015. Patients who met the following criteria were included in this study: (1) adults aged 18 years or older; (2) patients with pathologically confirmed NSCLC (9); their histologic types selected were coded as 8012/3,8013/3, 8022/3, 8031/3, 8032/3, 8033/3, 8035/3, 8046/3, 8050/3, 8052/3, 8070/3, 8071/3, 8072/3, 8074/3, 8082/3, 8083/3, 8084/3, 8123/3, 8140/3,8200/3, 8201/3, 8250/3, 8252/3, 8253/3, 8255/3, 8260/3, 8310/3, 8323/3, 8430/3, 8480/3,8481/3, 8490/3, 8550/3, 8560/3, 8570/3, 8574/3, and 8980/3; (3) patients who were diagnosed with stage IIIA-N2 NSCLC according to the guidelines of the American Joint Committee on Cancer (AJCC, 7th Edition); (4) NSCLC patients who had underwent either lobectomy or pneumonectomy; (5) the number of lymphadenectomy and positive lymph node metastasis was recorded after surgical operation; and (6) complete radiotherapy information record (patients who received postoperative adjuvant radiotherapy or did not receive radiotherapy).

Patients with the following conditions were excluded from this study: (1) patients with incomplete information registration required by the research; and (2) patients whose survival time was <1 month.

Variables extracted from the SEER database include the following: age at diagnosis, year of diagnosis, sex, race recode, primary site (main bronchus, upper lobe, middle lobe, lower lobe, and overlapping lesion of lung), International Classification of Diseases for Oncology (ICD-O) 3 Hist/behave, grade, derived AJCC T, RX summ-surg prim site, regional nodes positive, radiation sequence with surgery, chemotherapy recode, survival months, vital status recode, COD to site recode, SEER cause-specific death classification, and SEER other cause of death classification.

For a better analysis, all variables are converted to categorical variables. The chi-square test was used to evaluate the unadjusted association between the PORT and other clinicopathological categorical variables of interest. The hazard ratio (HR) was determined by univariate and multivariate Cox proportional hazard models. The aforementioned statistical calculations were carried out using SPSS 19.0 software. Overall survival (OS) was defined as the time from the beginning of the diagnosis until death of any cause or until the last follow-up date. The survival analysis was performed using Kaplan–Meier curves with *P* value determined by log-rank method. Figures of survival curve were drawn by “ggplot2,” “survminer,” and “survival” packages in R; the version of R was 3.6.0. All statistical tests were two-sided, and *P* < 0.05 was considered statistically significant.

## Results

### The Correlation Between Clinical Parameters and Postoperative Radiotherapy Use of IIIA-N2 Non-small Cell Lung Cancer Patients

**Figure 1:**
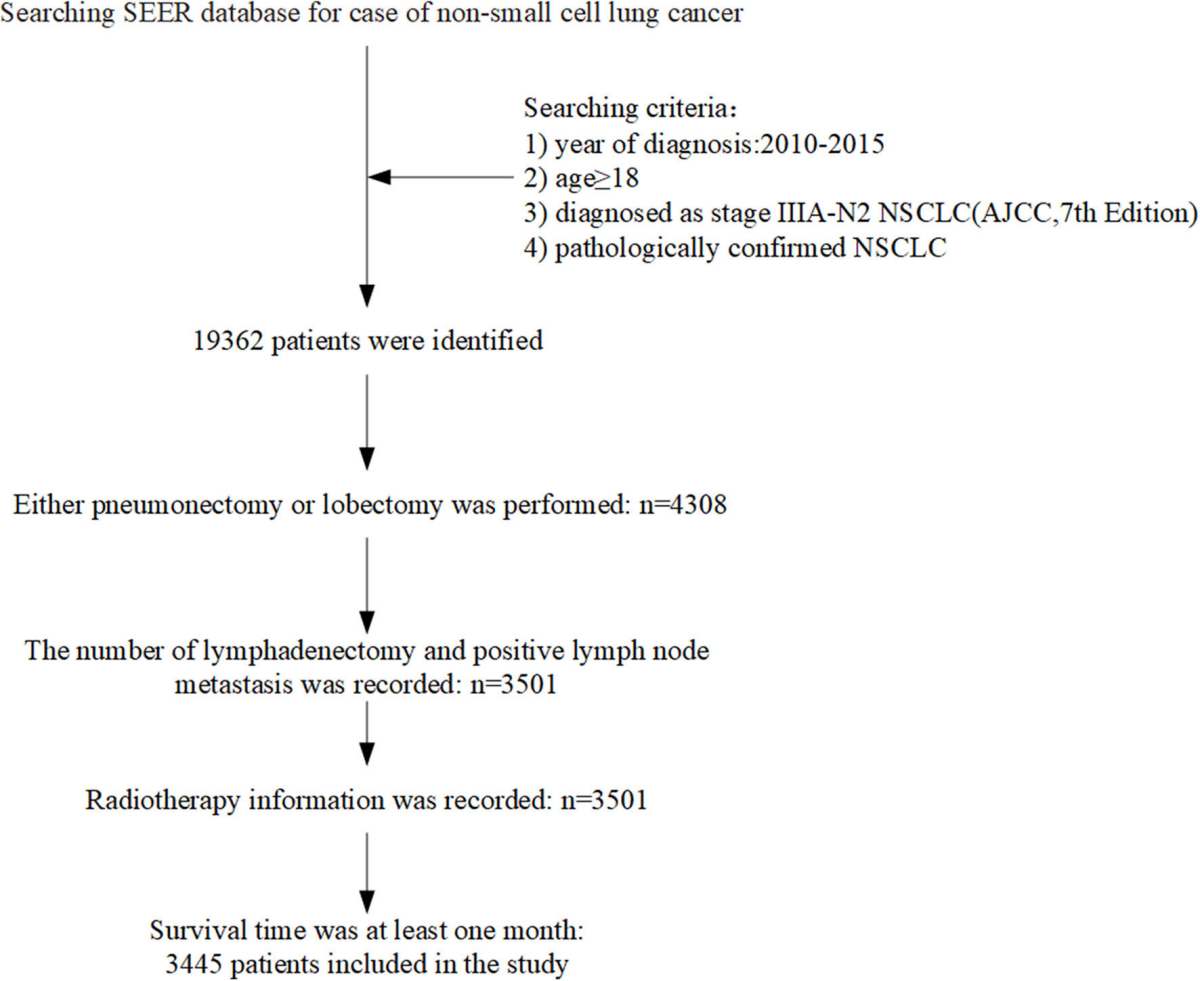
Flow chart showed selection of non-small cell lung cancer (NSCLC) patients registered in the SEER (Surveillance, Epidemiology, and End Results) from 2010 to 2015 in this study.

We identified 220,265 lung cancer patients registered in 2010–2015. According to the inclusion criteria, 3,445 patients were included in this study. Figure 1 showed the flow chart of cases selection. The median age at diagnosis was 67 years (age ranging from 19 to 91). A total of 1,568 (45.52%) patients received PORT. The proportion of patients who received radiotherapy differed in age, primary site of tumors, pathological grading of tumors, and whether they were treated with chemotherapy (*P* < 0.05). There was no significant difference in gender, year of diagnosis, race, T stage, number of positive regional lymph nodes, and pathological type between patients who received PORT and those not received PORT. The results are shown in Table 1.

**Table 1:** The correlation between clinical parameters and PORT use.

**Clinical parameters**	**No. of patients who did not receive PORT**	**No. of Patients who received PORT**	**No. of patients**	***P***
Age at diagnosis				<0.001
<60	367 (19.6%)	467 (29.8%)	834 (24.2%)	
≥60	1,510 (80.4%)	1,101 (70.2%)	2,611 (75.8%)	
Gender				0.532
Male	934 (49.8%)	797 (50.8%)	1,731 (50.2%)	
Female	943 (50.2%)	771 (49.2%)	1,714 (49.8%)	
Race				0.565
Black	186 (9.9%)	139 (8.9%)	325 (9.4%)	
White	1,534 (81.7%)	1,293 (82.5%)	2,827 (82.1%)	
Others	157 (8.4%)	136 (8.7%)	293 (8.5%)	
Year of diagnosis				0.584
2010	345 (18.4%)	272 (17.3%)	617 (17.9%)	
2011	333 (17.7%)	261 (16.6%)	594 (17.2%)	
2012	309 (16.5%)	277 (17.7%)	586 (17.0%)	
2013	307 (16.4%)	238 (15.2%)	545 (15.8%)	
2014	291 (15.5%)	260 (16.6%)	551 (16.0%)	
2015	292 (15.6%)	260 (16.6%)	552 (16.0%)	
Primary tumor site				0.004
Main bronchus	16 (0.9%)	18 (1.1%)	34 (1.0%)	
Upper lobe	1,045 (55.7%)	921 (58.7%)	1,966 (57.1%)	
Middle lobe	82 (4.4%)	93 (5.9%)	175 (5.1%)	
Lower lobe	671 (35.7%)	508 (32.4%)	1,179 (34.2%)	
Overlapping lesion	47 (2.5%)	21 (1.3%)	68 (2.0%)	
Unknown	16 (0.9%)	7 (0.4%)	23 (0.7%)	
Pathology				0.755
Adenocarcinoma	1,359 (72.4%)	1,153 (73.5%)	2,512 (72.9%)	
Squamous cell	417 (22.2%)	333 (21.2%)	750 (21.8%)	
Others	101 (5.4%)	82 (5.2%)	183 (5.3%)	
Pathological grade				<0.001
I	111 (5.9%)	67 (4.3%)	178 (5.2%)	
II	802 (42.7%)	630 (40.2%)	1,432 (41.6%)	
III	825 (44.0%)	675 (43.0%)	1,500 (43.5%)	
IV	24 (1.3%)	24 (1.5%)	48 (1.4%)	
Unknown	115 (6.1%)	172 (11.0%)	287 (8.3%)	
T				0.320
T1	510 (27.2%)	410 (26.1%)	920 (26.7%)	
T2	1,001 (53.3%)	820 (52.3%)	1,821 (52.9%)	
T3	366 (19.5%)	338 (21.6%)	704 (20.4%)	
POCT				<0.001
No	726 (38.7%)	101 (6.4%)	827 (24.0%)	
Yes	1,151 (61.3%)	1,467 (93.6%)	2,618 (76.0%)	
No. of positive lymph nodes				0.053
<6	1,513 (80.6%)	1,222 (77.9%)	2,735 (79.4%)	
≥6	364 (19.4%)	346 (22.1%)	710 (20.6%)	

*Pathological grade I, well-differentiated; II, moderately differentiated; III, poorly differentiated; IV, undifferentiated, anaplastic*.

*PORT, postoperative radiotherapy; POCT, postoperative chemotherapy*.

### Univariate Analysis of Clinical Parameters Affecting the Prognosis of IIIA-N2 Non-small Cell Lung Cancer Patients

PORT and POCT were favorable prognostic factors and associated with better OS in univariate analyses of all IIIA-N2 patients. Adverse prognostic factors included age (≥60 years old), male, non-adenocarcinoma pathological type, higher T stage, and ≥6 number of positive regional lymph nodes. We divided all the patients into two groups according to the number of positive lymph node metastases. The previous prognostic factors were consistent in a univariate survival analysis of IIIA-N2 NSCLC patients with <6 positive lymph node metastases, whereas the pathological type was not the prognostic factor in patients with ≥6 positive lymph node metastases. The results are shown in Table 2.

**Table 2:** Univariate analysis of clinical parameters affecting the prognosis of IIIA-N2 NSCLC patients.

**Parameters**	**All patients (*****n*** **= 3,445)**			**No. of patients with <6 positive lymph nodes (*****n*** **= 2,735)**			**No. of patients with≥ 6 positive lymph nodes (*****n*** **= 710)**		
	**HR**	**95% CI**	***P***	**HR**	**95% CI**	***P***	**HR**	**95% CI**	***P***
PORT									
No	1.00 (Ref)			1.00 (Ref)			1.00 (Ref)		
Yes	0.793	0.715–0.881	<0.001	0.836	0.741–0.943	0.004	0.623	0.506–0.766	<0.001
Age at diagnosis									
<60	1.00 (Ref)			1.00 (Ref)			1.00 (Ref)		
≥60	1.497	1.316–1.702	<0.001	1.535	1.319–1.786	<0.001	1.492	1.169–1.903	<0.001
Gender									
Male	1.00 (Ref)			1.00 (Ref)			1.00 (Ref)		
Female	0.671	0.605–0.745	<0.001	0.641	0.568–0.723	<0.001	0.791	0.644–0.972	0.026
Race									
Black	1.00 (Ref)			1.00 (Ref)			1.00 (Ref)		
White	1.180	0.983–1.416	0.076	1.215	0.979–1.507	0.077	1.137	0.804–1.607	0.467
Others	0.902	0.649–1.172	0.438	0.846	0.618–1.158	0.296	1.067	0.663–1.718	0.789
Year of diagnosis									
2010	1.00 (Ref)			1.00 (Ref)			1.00 (Ref)		
2011	1.031	0.892–1.191	0.682	1.085	0.916–1.283	0.345	0.898	0.676–1.192	0.456
2012	0.994	0.853–1.158	0.939	1.056	0.884–1.262	0.545	0.848	0.629–1.145	0.282
2013	0.928	0.782–1.102	0.397	0.985	0.808–1.200	0.878	0.847	0.598–1.199	0.348
2014	0.849	0.691–1.044	0.121	0.862	0.075–1.100	0.232	0.828	0.561–1.220	0.339
2015	0.800	0.565–1.133	0.208	0.801	0.527–1.217	0.298	0.770	0.412–1.439	0.413
Primary tumor site									
Main bronchus	1.00 (Ref)			1.00 (Ref)			1.00 (Ref)		
Upper lobe	0.833	0.491–1.412	0.479	0.930	0.463–1.869	0.839	0.840	0.372–1.893	0.673
Middle lobe	0.837	0.471–1.488	0.544	0.950	0.450–2.007	0.894	0.737	0.294–1.846	0.515
Lower lobe	1.039	0.611–1.767	0.887	1.168	0.580–2.353	0.663	0.973	0.430–2.204	0.948
Overlapping lesion	1.259	0.679–2.334	0.465	1.218	0.547–2.711	0.630	1.947	0.729–5.196	0.183
Unknown	0.933	0.414–2.100	0.867	1.642	0.634–4.257	0.307	0.169	0.020–1.407	0.100
Pathology									
Adenocarcinoma	1.00 (Ref)			1.00 (Ref)			1.00 (Ref)		
Squamous cell	1.336	1.184–1.507	<0.001	1.419	1.238–1.625	<0.001	1.227	0.943–1.597	0.127
Others	1.367	1.106–1.691	0.004	1.474	1.165–1.865	0.001	1.177	0.711–1.949	0.526
Pathological grade									
I	1.00 (Ref)			1.00 (Ref)			1.00 (Ref)		
II	0.978	0.766–1.247	0.855	1.023	0.774–1.352	0.873	0.751	0.453–1.244	0.266
III	1.247	0.980–1.587	0.073	1.266	0.961–1.668	0.093	1.045	0.636–1.719	0.861
IV	1.513	0.954–2.399	0.078	1.664	0.979–2.831	0.060	0.936	0.369–2.377	0.889
Unknown	0.945	0.694–1.286	0.718	0.878	0.614–1.255	0.474	1.213	0.655–2.247	0.539
T									
T1	1.00 (Ref)			1.00 (Ref)			1.00 (Ref)		
T2	1.220	1.073–1.386	0.002	1.220	1.055–1.411	0.007	1.092	0.835–1.429	0.519
T3	1.640	1.412–1.905	<0.001	1.640	1.381–1.949	<0.001	1.432	1.056–1.942	0.021
POCT									
No	1.00 (Ref)			1.00 (Ref)			1.00 (Ref)		
Yes	0.558	0.499–0.624	<0.001	0.561	0.494–0.638	<0.001	0.503	0.401–0.631	<0.001
No. of positive lymph nodes									
<6	1.00 (Ref)			——			——		
≥6	1.556	1.382–1.752	<0.001	——	——	——	——	——	——

*Pathological grade I, well differentiated; II, moderately differentiated; III, poorly differentiated; IV, undifferentiated, anaplastic*.

*HR, hazard ratio; Ref, reference; PORT, postoperative radiotherapy; POCT, postoperative chemotherapy; NSCLC, non-small cell lung cancer*.

### Multivariate Analysis of Clinical Parameters Affecting the Prognosis of IIIA-N2 Non-small Cell Lung Cancer Patients

The multivariate survival analysis of all patients showed that age (≥60 years), being male, non-adenocarcinoma pathological type, higher T stage, and ≥6 number of positive regional lymph nodes were independent risk factors for prognosis, indicating a shorter survival period. POCT were favorable prognostic factors and related to longer survival period.

We conducted a subgroup multivariate survival analysis according to the number of positive lymph node metastases (<6 positive lymph nodes and ≥6 positive lymph nodes). In a group of patients with <6 positive lymph node metastases, the results showed that PORT had no significant impact on survival (HR = 1.012, *P* = 0.858, 95% CI: 0.886–1.156). POCT had a positive effect on survival in IIIA-N2 patients with <6 positive lymph node metastases (HR = 0.573, *P* < 0.001, 95% CI: 0.498–0.660). Chemotherapy can prolong the survival of patients. The multivariate survival analysis showed that POCT (HR = 0.605, *P* < 0.001, 95% CI: 0.468–0.783) and PORT (HR = 0.724, *P* = 0.007, 95% CI: 0.574–0.914) are both favorable prognostic factors for stage IIIA-N2 patients with ≥6 positive lymph nodes; POCT and PORT can significantly improve OS. The multivariate analysis results are shown in Table 3.

**Table 3:** Multivariate analysis of clinical parameters affecting the prognosis of IIIA-N2 NSCLC patients.

**Parameters**	**All patients (*****n*** **=** **3,445)**			**No. of patients with <6 positive lymph nodes (*****n*** **=** **2,735)**			**No. of patients with** **≥6 positive lymph nodes (*****n*** **=** **710)**		
	**HR**	**95% CI**	***P***	**HR**	**95% CI**	***P***	**HR**	**95% CI**	***P***
PORT									
No	1.00 (Ref)			1.00 (Ref)			1.00 (Ref)		
Yes	0.931	0.830–1.045	0.226	1.012	0.886–1.156	0.858	0.724	0.574–0.914	0.007
Age at diagnosis									
<60	1.00 (Ref)			1.00 (Ref)			1.00 (Ref)		
≥60	1.322	1.158–1.510	<0.001	1.303	1.114–1.524	0.001	1.356	1.051–1.748	0.019
Gender									
Male	1.00 (Ref)			1.00 (Ref)			1.00 (Ref)		
Female	0.696	0.626–0.775	<0.001	0.667	0.589–0.755	<0.001	0.776	0.627–0.961	0.020
Race									
Black	1.00 (Ref)			1.00 (Ref)			1.00 (Ref)		
White	1.091	0.907–1.312	0.357	1.104	0.889–1.372	0.371	1.090	0.765–1.553	0.632
Other	0.833	0.639–1.085	0.175	0.757	0.551–1.039	0.085	1.084	0.661–1.777	0.749
Year of diagnosis									
2010	1.00 (Ref)			1.00 (Ref)			1.00 (Ref)		
2011	1.028	0.888–1.189	0.713	1.039	0.877–1.232	0.657	0.971	0.725–1.301	0.844
2012	1.026	0.880–1.196	0.745	1.066	0.891–1.276	0.483	0.943	0.693–1.281	0.706
2013	0.964	0.810–1.146	0.675	0.987	0.808–1.206	0.900	0.884	0.619–1.264	0.500
2014	0.871	0.708–1.073	0.194	0.870	0.680–1.111	0.264	0.846	0.572–1.253	0.404
2015	0.799	0.564–1.132	0.207	0.819	0.538–1.246	0.351	0.772	0.413–1.444	0.418
Primary tumor site									
Main bronchus	1.00 (Ref)			1.00 (Ref)			1.00 (Ref)		
Upper lobe	1.073	0.628–1.833	0.797	1.232	0.608–2.498	0.563	0.857	0.372–1.976	0.717
Middle lobe	1.119	0.624–2.006	0.705	1.280	0.600–2.734	0.523	0.831	0.325–2.125	0.699
Lower lobe	1.274	0.744–2.181	0.377	1.461	0.719–2.968	0.295	1.016	0.439–2.353	0.970
Overlapping lesion	1.487	0.799–2.768	0.211	1.523	0.680–3.411	0.306	1.663	0.609–4.536	0.321
Unknown	0.892	0.394–2.020	0.784	1.926	0.737–5.035	0.181	0.148	0.017–1.251	0.079
Pathology									
Adenocarcinoma	1.00 (Ref)			1.00 (Ref)			1.00 (Ref)		
Squamous cell	1.159	1.022–1.316	0.022	1.194	1.035–1.378	0.015	1.037	0.784–1.371	0.800
Others	1.244	0.994–1.558	0.057	1.296	1.009–1.664	0.043	0.999	0.587–1.698	0.996
Pathological grade									
I	1.00 (Ref)			1.00 (Ref)			1.00 (Ref)		
II	0.960	0.750–1.228	0.745	0.950	0.717–1.259	0.722	0.978	0.580–1.650	0.934
III	1.159	0.906–1.483	0.239	1.119	0.845–1.482	0.432	1.291	0.764–2.183	0.341
IV	1.211	0.752–1.950	0.432	1.323	0.763–2.297	0.319	1.021	0.382–2.729	0.967
Unknown	1.029	0.749–1.412	0.861	0.891	0.617–1.285	0.536	1.516	0.790–2.906	0.211
T									
T1	1.00 (Ref)			1.00 (Ref)			1.00 (Ref)		
T2	1.161	1.020–1.322	0.024	1.210	1.044–1.403	0.011	1.021	0.773–1.347	0.886
T3	1.492	1.280–1.738	<0.001	1.531	1.284–1.825	<0.001	1.420	1.035–1.947	0.030
POCT									
No	1.00 (Ref)			1.00 (Ref)			1.00 (Ref)		
Yes	0.573	0.507–0.648	<0.001	0.573	0.498–0.660	<0.001	0.605	0.468–0.783	<0.001
No. of positive lymph nodes									
<6	1.00 (Ref)			——			——		
≥6	1.602	1.420–1.807	<0.001	——	——	——	——	——	——

*Pathological grade I, well-differentiated; II, moderately differentiated; III, poorly differentiated; IV, undifferentiated, anaplastic*.

*HR, hazard ratio; Ref, reference; PORT, post operative radiotherapy; POCT, postoperative chemotherapy; NSCLC, non-small cell lung cancer*.

### Effect of Postoperative Radiotherapy on Overall Survival of IIIA-N2 Patients Divided by Number of Positive Lymph Node Metastases

**Figure 2:**
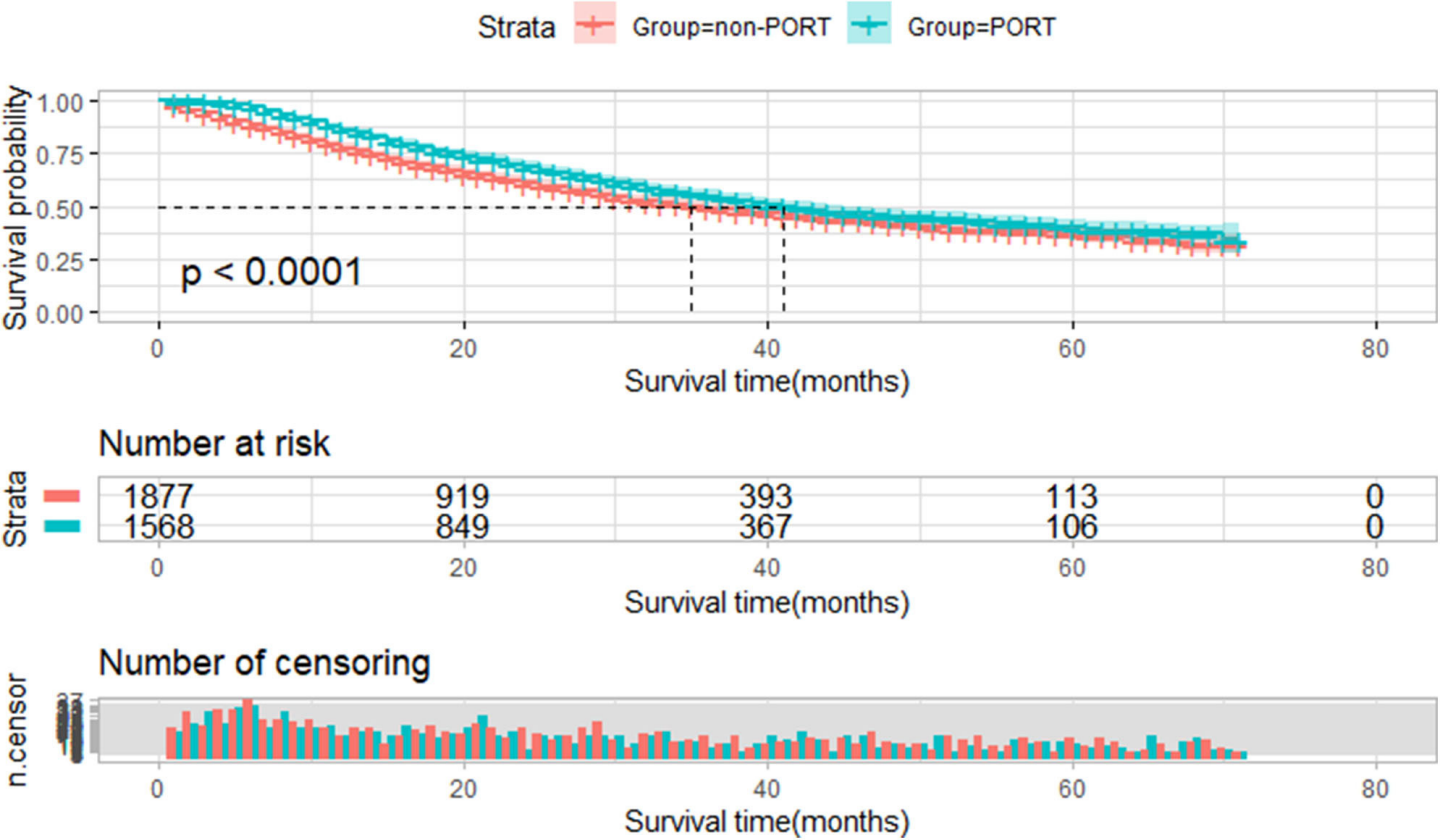
Overall survival of all selected patients stratified by postoperative radiotherapy (PORT). Overall survival of all patients treated with PORT (*n* = 1,568) vs. patients not treated with PORT (*n* = 1,877) (*P* < 0.001).

Among 3,445 IIIA-N2 NSCLC patients, PORT showed a statistically significant survival advantage relative to non-PORT (41 vs. 35 months, *P* < 0.001, Figure 2).

**Figure 3:**
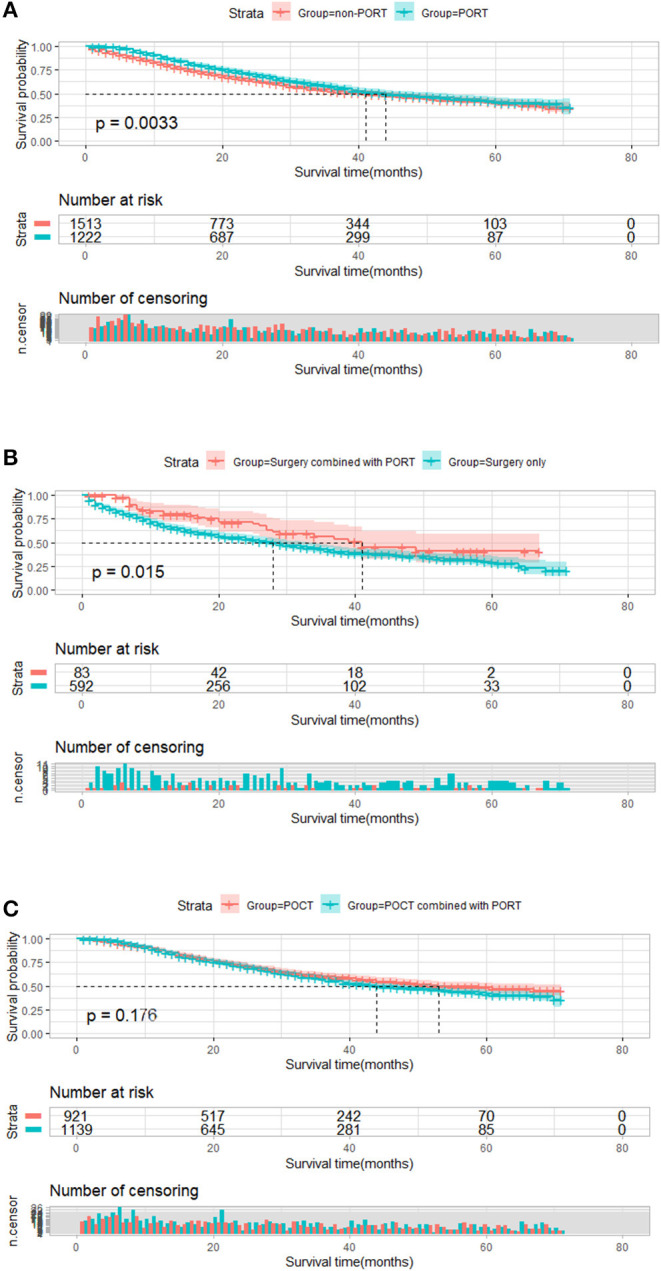
Overall survival of patients who featured <6 number of positive regional lymph node metastasis stratified by postoperative radiotherapy (PORT). **(A)** Overall survival of patients who featured <6 number of positive regional lymph node metastasis treated with PORT (*n* = 1,222) vs. patients not treated with PORT (*n* = 1,513) (*P* = 0.003). **(B)** Overall survival of patients who featured <6 number of positive regional lymph node metastasis treated with surgery combined with PORT (*n* = 83) vs. patients treated with surgery only (*n* = 592) (*P* = 0.015). **(C)** Plot of overall survival for 2,060 patients who featured <6 number of positive regional lymph node metastasis received postoperative chemotherapy (POCT) stratified by PORT use. Patients who received POCT combined with PORT (*n* = 1,139) vs. patients who received POCT (*n* = 921) (*P* = 0.176).

A total of 2,735 patients who featured <6 number of positive regional lymph node metastasis were divided into two groups by PORT. We compared the survival differences between the two groups. There was a significant statistical difference in survival between these two groups; median survival time of patients with PORT was 44 months, longer than the median survival time of those without PORT (41 months, *P* = 0.003, Figure 3A). We further conducted a subgroup analysis to explore the impact of PORT on OS. In 2,735 patients without POCT, patients who received PORT had better survival than patients who underwent surgery treatment only (41 vs. 28 months, *P* = 0.015, Figure 3B). In 2,060 patients with POCT, there was no significant difference in the survival of postoperative patients who underwent POCT in view of whether received PORT; although the result was not statistically significant, the survival of patients who received POCT combined with PORT seemed to be worse than that of patients who received POCT (44 vs. 53 months, *P* = 0.176, Figure 3C).

**Figure 4:**
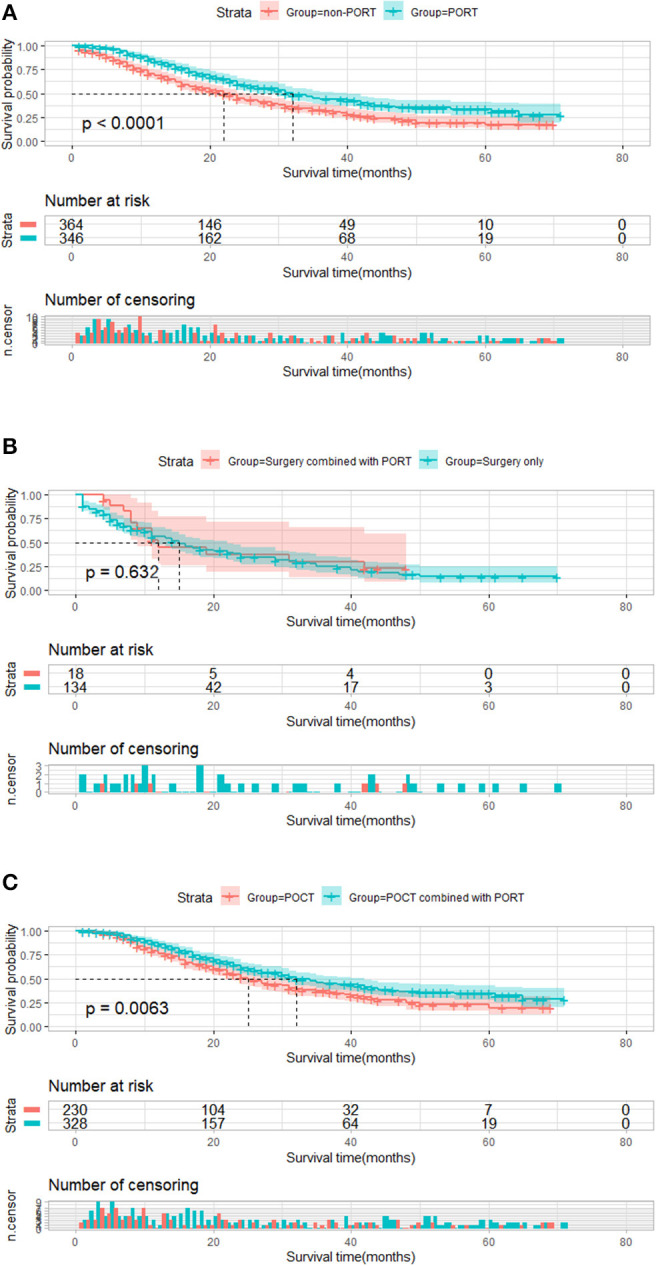
Overall survival of patients who featured ≥6 number of positive regional lymph node metastasis stratified by postoperative radiotherapy (PORT). **(A)** Overall survival of patients who featured ≥6 number of positive regional lymph node metastasis treated with PORT (*n* = 346) vs. patients not treated with PORT (*n* = 364) (*P* < 0.001). **(B)** Overall survival of patients who featured ≥6 number of positive regional lymph node metastasis treated with surgery combined with PORT (*n* = 18) vs. patients treated with surgery only (*n* = 134) (*P* = 0.632). **(C)** Plot of overall survival for 558 patients who featured ≥6 number of positive regional lymph node metastasis received postoperative chemotherapy (POCT) stratified by PORT use. Patients who received POCT combined with PORT (*n* = 328) vs. patients who received POCT (*n* = 230) (*P* = 0.0063).

Similarly, 710 patients who featured ≥6 number of positive regional lymph node metastasis were divided into two groups by PORT. Compared with that of patients who received PORT, the median survival time of patients who did not receive PORT was significantly shortened (32 vs. 22 months, *P* < 0.001, Figure 4A). The result of a subgroup analysis showed that for 152 patients without POCT, PORT did not prolong survival for postoperative patients who did not receive chemotherapy (12 vs. 15 months, *P* = 0.632). Kaplan–Meier plot is presented in Figure 4B. Among the 328 patients who received PORT combined with POCT and 230 patients who received POCT, PORT showed a significant advantage in influencing OS in these patients compared with those who received POCT only (32 vs. 25 months, *P* = 0.006). The result is shown Figure 4C.

### Overall Mortality of IIIA-N2 Patients Treated With Different Therapy

**Figure 5:**
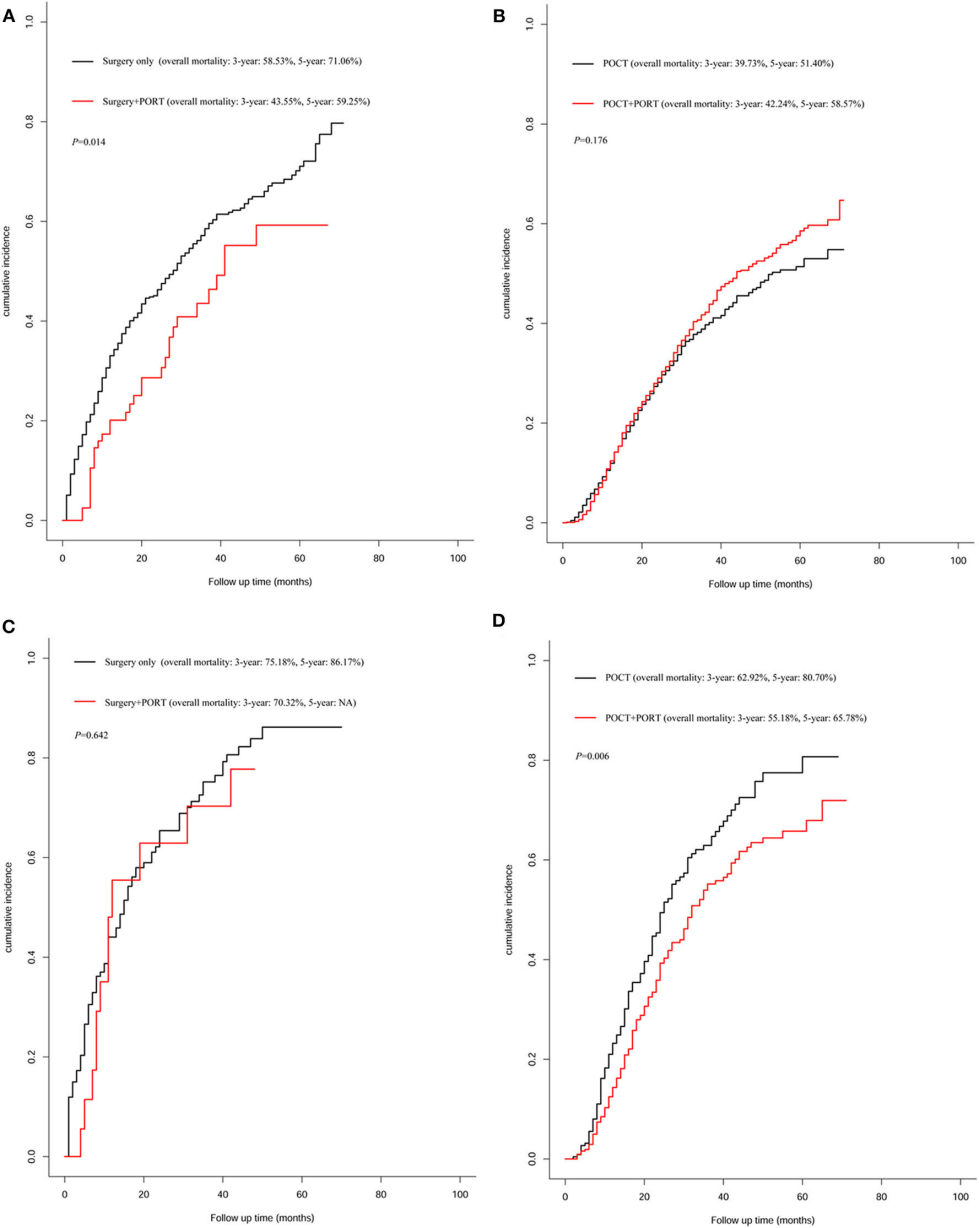
Analysis for overall mortality of patients treated with different therapy. **(A)** Overall mortality of patients who featured <6 number of positive regional lymph node metastasis treated with surgery combined with postoperative radiotherapy (PORT) vs. patients treated with surgery only (*P* = 0.014). **(B)** Overall mortality of patients who featured <6 number of positive regional lymph node metastasis received postoperative chemotherapy (POCT) combined with PORT vs. patients who received POCT (*P* = 0.176). **(C)** Overall mortality of patients who featured ≥6 number of positive regional lymph node metastasis treated with surgery combined with PORT vs. patients treated with surgery only (*P* = 0.642). **(D)** Overall mortality of patients who featured ≥6 number of positive regional lymph node metastasis received POCT combined with PORT vs. patients who received POCT (*P* = 0.006).

We analyzed the death outcomes of IIIA-N2 patients stratified by number of positive regional lymph nodes. In the group of 2,735 patients who featured <6 number of positive regional lymph node metastasis, the 3 and 5-years overall mortality rates of patients treated with surgery combined with PORT were significantly lower than those of patients treated with surgery alone (*P* = 0.014); the 3 and 5-years overall mortality rates of patients treated with surgery combined with PORT were 43.55 and 59.25%, respectively; and in patients treated with surgery alone, these rates were 58.53% and 71.06% (Figure 5A). Compared with patients who received POCT, the 3 and 5-years overall mortality rates were not different in patients treated with POCT combined with PORT (*P* = 0.176, Figure 5B).

In the group of 710 patients who featured ≥6 number of positive regional lymph node metastasis, PORT did not reduce overall mortality compared with mortality of patients who received surgery alone (*P* = 0.642, Figure 5C). For patients who received POCT, PORT can significantly reduce mortality; the result showed that the 3-years mortality rate decreased by 7.74% and the 5-years mortality rate decreased by 14.92% caused by PORT (*P* = 0.006, Figure 5D).

## Discussion

Stage IIIA-N2 NSCLC is a highly heterogeneous disease. The survival rate of stage IIIA-N2 patients after radical surgery was varied in different reported literatures. Even after complete resection, nearly 30% patients will suffer local recurrence or regional lymph node metastasis within 5 years (10). POCT can improve the disease-free survival (DFS) and OS by killing the local residual lesions and micrometastasis. National Comprehensive Cancer Network (NCCN) guidelines recommended POCT as a standard treatment for IIIA-N2 NSCLC patients. PORT, theoretically, can kill the residual tumor cells in the surgical field and significantly reduce the local recurrence rate of IIIA-N2 patients. The impact of the number of positive lymph nodes on the resected IIIA-N2 prognosis has been confirmed by many studies. Different N2 situations determine different prognoses and different treatment strategies. However, owing to the lack of research on the effect of PORT on NSCLC patients with stage IIIA-N2, it is still controversial whether PORT can bring OS benefits to IIIA-N2 patients. And also, owing to the lack of positive lymph node metastasis site records in SEER database and space limitation, we decided to stratify the analysis only for the number of positive nodes. Our study attempted to answer the clinical question: Does the state of N2 affect the PORT effect?

The largest postoperative radiotherapy meta-analysis of NSCLC conducted by PORT Meta-analysis Trialists Group revealed that the effect of PORT on IIIA-N2 patients was not clear (11). Kim et al. (12) evaluated the effect of PORT on stage III A-N2 NSCLC, and they found that PORT significantly improved the local control rate of stage III A-N2 NSCLC but did not prolong the OS period. But several studies have confirmed that PORT can prolong survival and reduce local recurrence in IIIA-N2 patients. Lally et al. (13) demonstrated that PORT increased the 5-years survival rate of pN2 patients from 20% to 27% and reduced the risk of death by 14.5%. The ANITA study (14) showed that PORT increased the 5-years survival rate of stage IIIA-N2 NSCLC from 34% to 47%. Herskovic et al. (15) showed that PORT could improve the survival of IIIA-N2 NSCLC patients, that PORT displayed a 17% reduced hazard of death as compared with non-PORT, and that the median overall survival was 53.1 months for PORT compared with 44.5 months for non-PORT. Based on the above different research conclusions, the 2017 version of American Society of Clinical Oncology (ASCO) guidelines does not recommend the routine use of adjuvant radiotherapy for IIIA-N2 patients; instead, the benefits and risks of adjuvant radiotherapy for each N2 patient should be evaluated in various aspects after operation. But the 2017 version of NCCN guidelines recommend that POCT or concurrent chemotherapy and radiotherapy should be used as adjuvant treatment for N2 patients who underwent complete resection. Corso et al. (16) retrospectively analyzed 30,552 patients with stage II-IIIA NSCLC who underwent R0 resection in the National Cancer Database (NCDB) from 1998 to 2006, 3,430 of whom received postoperative radiotherapy. The results revealed that the 5-years survival rate of stage N2 patients treated with PORT was improved (27.8 vs. 34.1%, *P* < 0.001), and the absolute survival rate was increased by 6.3% compared with that of patients who did not receive PORT. Sakib et al. (17) showed that PORT could significantly improve local control rate and survival of resected stage IIIA-N2 NSCLC patients, regardless of whether they received chemotherapy.

We included surgically resected IIIA-N2 lung cancer patients in the study, and the exact removed number of lymph nodes in all patients was recorded. All postoperative patients met the definition of N2 in the AJCC 7th Edition in SEER database. Therefore, the definition of N2 is not a clinical diagnosis but a postoperative confirmation.

Our results demonstrated for IIIA-N2 NSCLC patients that the survival time of patients who received PORT is significantly longer than that of non-PORT patients, which was similar to the previous research conclusion. However, the survival time of IIIA-N2 NSCLC patients was significantly different, and the number of lymph node metastasis was related to the prognosis of NSCLC. Can all N2 patients benefit from PORT regardless of number of lymph node metastases? Should PORT be a necessary and conventional treatment for all IIIA-N2 patients? We stratified the N2 patients according to the number of lymph node metastases and analyzed the benefits of PORT in different groups.

For patients with < six lymph node metastases, median survival of patients treated with surgery alone was 28 months, and the median survival of patients who received PORT treatment after radical surgery was extended to 41 months. PORT could prolong survival time as compared with no adjuvant therapy after surgery if these patients did not undergo POCT for some reason. PORT reduced the 3 and 5-years mortality rates by 14.98 and 11.81%, respectively. However, for patients with ≥6 number of positive regional lymph nodes metastasis, if they do not receive POCT, PORT did not improve survival and did not reduce mortality. We speculated that PORT can be used as an effective supplement treatment to surgery for patients with <6 lymph node metastases. Compared with surgery alone, use of PORT could be converted into survival benefits by reducing the local recurrence rate. Therefore, we suggested that for patients who cannot tolerate POCT, if their physical condition permits, PORT could be used as a recommended therapy.

Previous studies have confirmed the necessity of POCT in resectable IIIA-N2 patients. Does PORT improve survival on the basis of chemotherapy? Mikell et al. (18) analyzed 2,115 patients with NSCLC in N2 stage who received POCT from 2004 to 2006 in NCDB database, and they concluded that compared with the control group, PORT can improve the 5-years survival rate (39.8 vs. 34.7%, *P* = 0.048). Lei et al. (19) showed that compared with POCT, PORT combined with POCT was beneficial to OS of IIIA-N2 NSCLC patients, but not to DFS. Our results revealed that for patients with <6 lymph node metastases, PORT combined with POCT therapy has no significant benefit compared with POCT. Although it is not statistically significant, the median survival time of PORT combined with POCT group seems to be shorter than that of POCT group. But for the patients with ≥6 number of positive regional lymph node metastasis, PORT combined with POCT therapy is necessary. Compared with the median survival time of 25 months in the chemotherapy group, the treatment of PORT combined with POCT can prolong OS and increase the median survival time to 32 months. Meanwhile, PORT combined with POCT therapy can reduce the 3 and 5-years mortality rates by 7.74% and 14.92%, respectively. We speculated that for IIIA-N2 patients with ≥6 positive lymph nodes, the risk of local recurrence and distant metastasis are higher than those of other IIIA patients, so surgery alone is not enough; local and systemic treatment should be strengthened to reduce recurrence and metastasis in order to prolong the survival period. PORT has a beneficial effect on survival by eliminating the local micrometastasis, reducing the local recurrence and cancer-related death rate. POCT can prevent the systemic micrometastasis and recurrence so as to improve the survival period.

Conclusions about the effect of PORT from previous studies were inconsistent, which may be related to the different states of IIIA-N2 patients, as well as different radiotherapy equipment and doses used in studies. Our findings suggested that IIIA-N2 patients should be carefully evaluated according to the number of lymph node metastases before PORT treatment. POCT was necessary and important for all IIIA-N2 NSCLC patients. For patients with <6 lymph node metastases, if patients cannot tolerate POCT, the use of PORT to improve survival can be encouraged. For IIIA-N2 patients with ≥6 positive lymph nodes, if the patients' physical conditions allow, PORT combined with POCT therapy should be applied, because PORT alone did not have survival benefit for this group. The results are shown in Table 4.

**Table 4:** Strategy for postoperative adjuvant treatment of resected stage III A-N2 NSCLC.

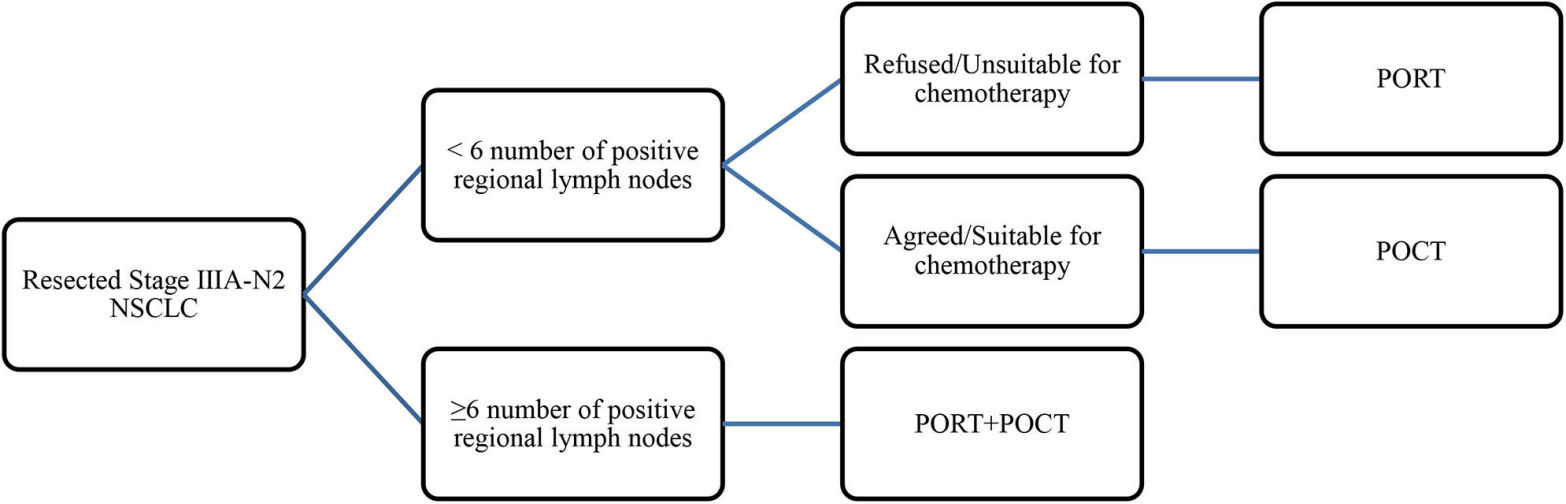

*NSCLC, non-small cell lung cancer*.

There are limitations in our research: We did not analyze the dose and range of radiotherapy owing to lack of record in SEER database. It is undeniable that our research was a retrospective study and bias was inevitable. We try to minimize this bias through a large data analysis and statistical method. At present, it is urgent to carry out a multicenter prospective randomized controlled study based on modern precise radiotherapy technology and unified radiotherapy program, so as to provide a higher level of evidence for the application of PORT in the stage IIIA-N2 NSCLC patients and guide the selection of the beneficiary population.

Currently, there is no standard treatment for locally advanced IIIA-N2 NSCLC, and the single treatment is limited. For resectable IIIA-N2 NSCLC, the chance of death from recurrence or metastasis within 5 years after operation is still high regardless of R0 resection. The optimal combination of surgery, chemotherapy, and radiotherapy treatment including perioperative target therapy and immunotherapy for patients with stage IIIA-N2 NSCLC is still to be determined. Each NSCLC patient with stage IIIA-N2 should be carefully evaluated by a multidisciplinary team to develop the best treatment strategy.

## Conclusion

In patients who featured <6 number of positive regional lymph nodes, patients who received PORT had better survival rate than patients who underwent surgery only. But in this group, there was no significant difference in the survival of postoperative patients who underwent POCT in view of whether received PORT. For patients with ≥6 positive lymph nodes, PORT combined with POCT significantly improved OS and decreased overall mortality. Owing to limitations in our study, a large-cohort, multicenter, and prospective study is needed.

## Data Availability Statement

The raw data supporting the conclusions of this article will be made available by the authors, without undue reservation.

## Ethics Statement

The studies involving human participants were reviewed and approved by This retrospective study was approved by Ethics Committee of Beijing Hospital of Traditional Chinese Medicine, Capital Medical University. Written informed consent for participation was not required for this study in accordance with the national legislation and the institutional requirements.

## Author Contributions

FG collected the data and wrote the original draft. NL provided statistical analysis. YX assisted in providing statistical analysis. GY provided the original idea of the manuscript and reviewed the manuscript. All authors contributed to the article and approved the submitted version.

## Conflict of Interest

The authors declare that the research was conducted in the absence of any commercial or financial relationships that could be construed as a potential conflict of interest.
